# Requirements for more effective prevention of work-related musculoskeletal disorders

**DOI:** 10.1186/s12891-015-0750-8

**Published:** 2015-10-14

**Authors:** Wendy Macdonald, Jodi Oakman

**Affiliations:** Centre for Ergonomics & Human Factors, School of Psychology and Public Health, La Trobe University, Bundoora, Victoria 3086 Australia

**Keywords:** Musculoskeletal disorders, Risk management, Work-related, Psychosocial, Manual handling, Hazards, Systems

## Abstract

**Background:**

Exposures to occupational hazards substantially increase workers’ risk of developing musculoskeletal disorders (MSDs) and can exacerbate pre-existing disorders. The effects on MSD risk of the physical requirements of work performance are well recognised, but there is now ample evidence that work-related psychosocial hazards can also have substantial effects; further, some hazards may be additive or interactive. This evidence is not reflected in current workplace risk management practices.

**Discussion:**

Barriers to more effective workplace management of MSD risk include: the widespread belief that risk arises largely or entirely from physical hazard exposures; regulatory and guidance documents targeting MSDs, most of which reflect this belief; risk assessment tools that focus narrowly on subsets of mainly physical hazards and yet generate outputs in the form of MSD risk indicators; and the conventional occupational health and safety (OHS) risk management paradigm, which is ill-suited to manage MSD risk.

It is argued that improved workplace management of MSD risk requires a systems-based management framework and more holistic risk assessment and control procedures that address risk from all types of hazard *together* rather than in isolation from each other, and that support participation by workers themselves. New MSD risk management tools are needed to meet these requirements.

Further, successful implementation of such changes is likely to require some restructuring of workplace responsibilities for MSD risk management. Line managers and supervisors often play key roles in generating hazards, both physical and psychosocial, so there is a need for their more active participation, along with OHS personnel and workers themselves, in routine risk assessment and control procedures.

**Summary:**

MSDs are one of our largest OHS problems, but workplace risk management procedures do not reflect current evidence concerning their work-related causes. Inadequate attention is given to assessing and controlling risk from psychosocial hazards, and the conventional risk management paradigm focuses too narrowly on risk from individual hazards rather than promoting the more holistic approach needed to manage the combined effects of all relevant hazards. Achievement of such changes requires new MSD risk management tools and better integration of the roles of OHS personnel with those of line managers.

## Background

The traditional occupational health and safety risk (OHS) management model is under strain as the burden shifts from injuries to illnesses arising from chronic disease [1]. This change is primarily due to the increasing proportion of occupational health problems that have complex, variable aetiologies – particularly musculoskeletal disorders (MSDs) and also mental health disorders. There are many non-work causes of MSDs, but exposure to occupational hazards is a major risk factor. For example, the World Health Organisation estimated that 37 percent of all back pain worldwide is attributable to work, resulting in an estimated 800,000 DALYs (disability-adjusted life years) lost [2]. Quantitative international comparisons are hindered by wide variation in OHS regulatory frameworks and data recording systems, but the prevalence and associated costs of work-related MSDs are very high throughout the industrially developed world, and are widely viewed as one of our largest OHS problems [3, 4]. The challenge is compounded as populations age, and in many countries there is an increasing economic need for people to continue working to older ages than currently [5, 6].

Unfortunately, current OHS risk management strategies targeting MSDs and associated risk control interventions fail to reflect the large body of research evidence that has identified the main work-related sources of this risk and the requirements for effective workplace interventions to reduce it.

### Work-related sources of MSD risk

Prior to the 1990s it appeared that work-related MSD risk arose largely or entirely from various hazards associated with the physical requirements of work performance, often referred to as manual handling hazards and sometimes as ‘ergonomic’ hazards. However, there is now an evidence-based consensus among researchers that MSD risk is *also* influenced by a diverse range of non-physical hazards, as outlined below. Importantly, the effects on MSD risk of many of these hazards have been shown to be additive or interactive (e.g. [7–9]). Focusing just on physical hazards, Marras and colleagues noted that “the impact of the interactions may be far greater than that of any individual factor” [10].

Based on an extensive review of research evidence, a landmark 2001 report [11] categorized work-related sources of MSD risk as: (a) external loads, here termed *physical hazards* (e.g. heavy lifting, repetitive actions; adverse postures); (b) organisational factors (e.g. high workloads, night shifts) and (c) social context (e.g. low supervisor support, low recognition). Organisational and social context factors together are here termed *psychosocial hazards*, consistent with terminology of the European Framework for Psychosocial Risk Management [12]. According to that Framework, psychosocial hazards include factors related to: job content, workload and work pace, work schedule, control, organisational culture and function, interpersonal relationships at work, role in organisation, career development, and home-work interface. Lang and colleagues [13] confirmed the *causal* impact of workplace psychosocial hazards on MSD risk via a systematic review and meta-analysis of results from a large set of baseline-adjusted prospective longitudinal studies, while Eatough and colleagues [8] demonstrated the role of resultant ‘psychological strain’ in mediating the effects of psychosocial hazards on MSD risk.

**Fig. 1:**
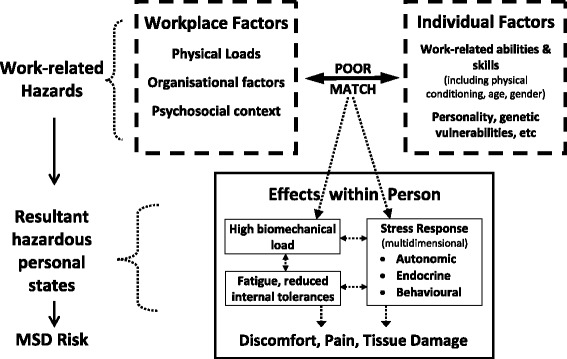
A simplified composite ‘model of causation’ for MSD risk [15]

Various theoretical models have been developed to depict the pathways connecting these diverse physical and psychosocial hazards to MSD risk (e.g. [11, 14, 15]). Some of these pathways involve internal tissue loads stemming from the biomechanical demands of manual task performance, while others involve various physiological concomitants of the multidimensional stress response [8, 10, 16–23]. Figure 1 presents a simplified composite model of causative factors [15].

The relative influence on risk of psychosocial versus physical hazards varies widely across different studies, but their influence is typically reported to be substantial [10, 13, 24–28]. Marras and colleagues [10] concluded from their review of evidence that:

between 11 and 80 % of low-back injuries and 11–95 % of extremity injuries, are attributable to workplace physical factors, whereas, between 14 and 63 % of injuries to the low back and between 28 and 84 % of injuries of the upper extremity are attributable to psychosocial factors

This large variation is probably due to differences between the studies in levels of workplace hazards and their associated interactions, as well as to varying hazard measurement methods. A recent prospective longitudinal study by Gerr and colleagues [27, 29] employed unusually good measures of both physical *and* psychosocial hazards affecting MSD risk (neck/shoulders, upper extremities) of manufacturing workers, and statistically controlled for a large set of potentially confounding variables. They analysed and reported results for physical and psychosocial hazards separately and did not discuss their comparative influence on risk, but it is noteworthy that hazard ratios (HRs) for physical hazard exposures were mostly very low and few were statistically significant, whereas many of the HRs for psychosocial hazards were high and most were significant.

Such evidence is important because hazard effect sizes, whether in terms of attributable fractions [10] or hazard ratios [27, 29] are useful indicators of the extent to which MSD risk in a particular work situation might be reduced if such hazards are reduced. Gerr and colleagues [27] concluded that management of psychosocial hazards needs to be an integral component of routine workplace risk management for MSDs.

### Current workplace approaches to MSD risk management

Documentation of actual workplace risk management practices is rare. Research in four large Australian workplaces found that minimal attention was given to psychosocial hazards, and in two of the four workplaces there was a major emphasis on training workers in ‘safe’ movement techniques [30], despite strong research evidence that this is *unlikely* to reduce MSD risk [31, 32]; more recent Australian research in the aged care sector found there is still minimal attention to MSD risk from psychosocial hazards [33]. In the UK, research on workplace MSD risk management practices of consultant ergonomists also found a narrow focus on physical hazards [34]. The authors noted that the ergonomists failed to take adequate account of the organisational context and work environment, and that their actions were constrained by workplace expectations that MSD risk should be assessed and controlled purely on the basis of physical hazard exposures. Such findings accord with widespread anecdotal evidence that MSD risk management practices are still largely uninfluenced by evidence of the substantial effects of psychosocial hazards.

## Discussion

Why is there such a large gap between research evidence and workplace practices? In the sections below, we identify some major barriers that are hindering both communication of the need for changes and workplace implementation of changes. We argue that overcoming these barriers requires expansion of the conventional OHS risk management paradigm, as well as new risk management tools to enable more holistic management of MSD risk arising from both psychosocial and physical hazards within a broader systems-based framework. Finally, some more general implications for workplace management are identified.

### Barriers to more effective risk management

#### Common misperceptions of MSD causation

A widespread assumption throughout the community is that *physical* disorders such as MSDs must be largely if not entirely caused by hazards arising from *physical* activities, while *psychosocial* hazards are seen as primarily affecting stress-related *psychological* health problems. This probably reflects the continuing influence of mind/body dualism on our thinking about health, which endures despite greater attention by medical practitioners to patients’ experiences and a strengthening of multi-causal views of disease [35]. Because of this apparently ‘common sense’ assumption, managers are likely to see implementation of risk management procedures targeting psychosocial hazards as unnecessary in workplaces where OHS costs relate largely to MSDs rather than mental health problems … particularly when this viewpoint is reinforced by the content of current OHS regulations and guidance targeting MSD risk.

#### Inadequacies of MSD risk management regulatory and guidance documents

Government regulatory bodies throughout the world continue to focus largely on the physical hazards affecting MSD risk. A 2003 content analysis of 33 MSD-related regulatory Standards, Codes of Practice and Guidance documents worldwide, selected as being English language and of the highest available quality, found very poor coverage of how to assess and control risk from relevant psychosocial hazards [36]. Despite further accumulation of research evidence on the substantial effects of psychosocial hazards on MSD risk, there appears to have been no improvement in their coverage, as outlined below.

In the UK, MSD risk management guidance on the website of the Health and Safety Executive provides extensive coverage of how to assess and control risk from the physical hazards associated with manual task performance, but no advice on how to assess risk from psychosocial hazards and little on controlling it [37]. Psychosocial hazards are mentioned only within a tool for assessing risk from repetitive tasks, where just a few hazards are listed and advice is confined to: “Psychosocial factors are not given a score. However, they should be considered and, if present in the workplace, recorded on the score sheet. Psychosocial factors should be considered through discussion with workers” [38].

The situation is much the same elsewhere. For example Australia’s 2011 *Hazardous Manual Tasks Code of Practice* specifies the first step in managing MSD risk as: “to identify those tasks that have the potential to cause MSDs” [39]. Consistent with its title, it narrows attention to particular tasks and their associated workstations, tools and equipment. In its eight pages on *Assessing the Risk,* less than half a page is allocated to psychosocial factors, which are listed as sources of risk within *Systems of Work*. There is no mention of how such risk could be assessed and minimal guidance on control strategies (approximately 1 page out of 15). Similarly in Canada, a recently developed toolkit intended for workplace use in preventing musculoskeletal disorders includes some reference to work organisation and work process and how problems might be identified, but relevant controls are not included despite extensive coverage of how to control risk from physical hazards [40].

A second limitation of current guidance materials arises from their structure being largely in accord with the conventional OHS risk management paradigm – that is, they address types of *hazard* (e.g. those arising from ‘hazardous manual tasks’), rather than types of *harmful outcome* (e.g. MSDs). This structuring into separate hazard-based categories is problematic, as discussed in the following section.

#### Inadequacies of the conventional OHS risk management paradigm

We argue that the conventional focus of OHS risk management on a type of hazard (e.g. biomechanical forces and associated postures) rather than a type of outcome such as MSDs and associated physical discomfort or pain [41] is an important barrier to more effective prevention of MSDs.

The conventional paradigm is well-suited for risks arising from exposures to hazardous substances or other forms of damaging energy such as electricity and loud noise that are unequivocally negative in their effects on health or safety [42]. However, this paradigm is not helpful when risk arises from the net effect of multiple and diverse hazards acting in variable combinations via complex causal pathways. For example, the level of force required to push a trolley might present a *low* MSD risk if exerted infrequently and a *high* risk if workers are exerting that force repetitively while also experiencing stress due to excessive time pressures or supervisors perceived as unsupportive. Given such complexities, assigning an MSD risk level and prioritising risk control measures based *only* on the severity of a small subset of hazards is unreliable, because it fails to take account of the possibly additive or interacting effects of other relevant hazards [43].

In this kind of situation the risk assessment process needs to be *holistic*; that is, it needs to consider risk and potential control measures for all hazards in combination. And because risk is affected by a large and diverse range of hazards arising variously from interactions between work task characteristics, work organisation, job design, psychosocial and physical environments and individual workers, the procedures to assess risk and select appropriate interventions to reduce it need to be supported by a broad *systems-based* conceptual framework or ‘model of causation’.

The need for this kind of systems-based risk management paradigm is now well accepted where there is risk of catastrophic accidents. For example in industries dealing with hazardous chemicals or nuclear power generation, the complex and highly variable pathways linking ‘hazards’ to potential major accidents have been well documented [44, 45]; the term ‘process safety’ has been applied to this kind of risk management paradigm and contrasted with the conventional OHS paradigm [46]. We argue that, rather than accept such a dichotomy, the OHS paradigm needs expansion to accommodate both conventional and systems-based approaches, so that MSD risk can be managed more effectively.

Another problem with the conventional paradigm is its ‘hierarchy of risk control’, where the aim is to identify and if possible *eliminate* a hazard or at least to reduce it as much as possible [47]. This hierarchy was originally developed for the control of traumatic injuries such as those to road vehicle occupants in crashes [42, 48]. In applying it to MSDs, the current Australian Code of Practice for Hazardous Manual Tasks states that: “Control measures should be aimed at eliminating or minimising the frequency, magnitude and duration of movements, forces and postures …” [39]. This lacks credibility since virtually all work performance inevitably entails *some* movements and force exertions, and eliminating or reducing them is not necessarily desirable because the health risks of sedentary work are now well established [49]. Much the same holds true for psychosocial hazards; for example, both very high and very low workloads can be hazardous [16, 50], so the aim should be to optimise rather than minimise [51, 52].

#### Inadequacies of MSD risk management tools

A great many tools have been developed for use in assessing MSD risk stemming from the physical aspects of work task performance, but no single tool currently covers all hazards, and there are substantial differences between tools in which hazards are addressed and how risk is assessed [53–57]. A few tools entail direct measurement of specific postures and movements (e.g. [58, 59]), but these are generally unsuitable for use by non-experts and would rarely be usable during routine workplace risk management. Most tools, including those most likely to be used by workplace risk managers, are based on observations of task performance. A comprehensive review of the validity and reliability of such observational tools found evidence of predictive validity (in terms of levels of MSD symptoms or diagnosed cases) for only 12 of the 32 tools examined, and for only 2 of the 12 was this evidence from longitudinal rather than cross-sectional studies [55].

There are several reasons for this very weak evidence of predictive validity. First, any one tool focuses on just a subset of physical hazards and none provides adequate coverage of psychosocial hazards, so they ignore many potential sources of risk. Second, the representativeness of task performance samples analysed by such tools is often dubious, partly because in many jobs it is common for workers to perform a variety of tasks for variable amounts of time, which makes it impracticable for *observers* to take adequate account of all physical exposures and their durations [29, 53, 56]. A possible solution would be to obtain such information from the workers themselves, as is sometimes done by researchers (e.g. [29, 60]).

#### Need for greater participation by workers in MSD risk assessment and control

Several systematic reviews have found that worker participation in MSD risk management tends to positively affect success [61–63]. Such participation is typically in the identification of hazards and/or the identification and implementation of related controls, rather than in risk assessment, and the extent to which it occurs in most workplaces is unknown. However, it was suggested above that in view of the weaknesses of existing tools, participation by workers in *assessing extent* of physical exposures could also be beneficial.

In the case of psychosocial hazards, worker participation in assessment is essential because many are not observable by others and their severity is strongly influenced by workers’ perceptions. In the case of *physical* hazards, participation by workers is often seen as unnecessary because the severity of such hazards is observable by others (at any particular time). Systematic procedures for obtaining such information from workers are currently not available, and the validity of self-reported data can lack credibility. However, a review of evidence on the criterion validity of worker ratings used in research studies found that when the two sets of information were appropriately matched, worker ratings were significantly correlated with data from reference methods [60]. It may be that for workplace risk managers, the *predictive* validity of hazard assessments relative to MSD outcomes is more important than criterion validity, and there is some evidence that subjective ratings can have greater predictive validity than observational tools [30], which is unsurprising in view of the limitations of such tools as outlined above.

### Pathways forward

#### Expansion of the OHS risk management paradigm

**Fig. 2:**
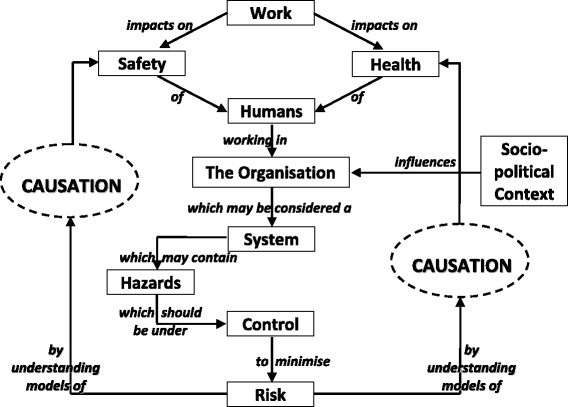
An expanded OHS risk management paradigm ([64], p. xviii)

We have argued that the conventional, hazard-focused risk management paradigm is satisfactory for many types of OHS risk, but effective management of MSD risk requires risk assessment and control procedures to consider all relevant hazards *together*, taking account of their potentially interactive effects. An expanded OHS risk management paradigm therefore needs to accommodate both types of approach. Figure 2 presents an example of such a paradigm, closely based on one formulated by the technical panel responsible for developing a ‘core body of knowledge’ for Australia’s generalist OHS professionals [64].

By specifying that effective risk control requires understanding of ‘models of causation’ – that is, models of the causal linkages between hazards and workers’ health or safety – this paradigm supports both the conventional hazard-focused approach and more holistic, systems-based approaches. For diseases and disorders that are linked directly to just *one* main work-related hazard, the model of causation is relatively simple and transparent (e.g. the model linking loud noise exposures with hearing loss); in such cases the conventional hazard-focused approach to risk management is appropriate. But for risks such as MSDs that are affected by exposures to multiple hazards in combinations that vary between different work situations, the model of causation is complex. In such cases effective risk management requires a systems-based paradigm, within which more holistic assessment and control procedures are able to take account of relationships between hazards, and between hazards and individual variables. The following section discusses the kinds of tools and associated resources needed to support workplace implementation of such a paradigm.

#### Tools and resources to support more holistic, systems-based MSD risk management

Work-related mental health disorders are similar to MSDs in that risk is affected by a diverse range of psychosocial hazards, and assessment and control procedures need to be holistic with a high level of participation by workers and their managers. It is therefore useful to look at the characteristics of tools and resources developed recently for workplace use in managing *mental* health risk [65–69]. Importantly, the procedures used in these tools are holistic; that is, all sources of risk to mental health are assessed and controlled together, with no attempt to identify ‘acceptable’ or ‘safe’ levels of any particular hazard or group of hazards in isolation from the others. Workers are actively involved both in risk assessment and in identification of potential risk control interventions.

It might seem reasonable simply to incorporate use of such tools within MSD risk management procedures, in parallel with tools targeting physical hazards. Unfortunately, these tools frequently refer to psychosocial hazards as psychosocial ‘risks’, which conflates hazard (relating to causes) with risk (relating to outcomes), thereby reinforcing the common misconception that psychosocial hazards are relevant only to mental rather than physical health problems; further, some of them explicitly target only psychological health outcomes. Given their focus on psychological health, it seems unlikely that they will be adopted in workplaces where OHS-related costs arise mainly from MSDs. Consequently, there is a need for tools that are clearly identified as targeting MSD risk, integrating management of risk from both physical and psychosocial hazards.

To this end, Macdonald, Oakman and colleagues have developed an MSDs risk management ‘toolkit’ (currently undergoing field trials) in accord with the World Health Organisation concept of a toolkit as a set of practical tools and strategies for workplace use in identifying hazards and assessing risk, and for developing, implementing and evaluating interventions to reduce risk [15, 26, 70]. Scores from worker ratings of discomfort/pain levels are used to indicate MSD risk for a target job; such scores have been validated in relation to the incidence of diagnosed MSD cases [27, 29, 41] and workers’ compensation claims [30, 33]. Psychosocial hazard levels are assessed by worker ratings from a standard survey – the Work Organisation Assessment Questionnaire [71], Physical hazards levels are similarly assessed by worker ratings of their own exposures to the physical hazards they encounter when performing all the tasks comprising their job; that is, assessment encompasses the overall job rather than focusing on specific tasks. The toolkit includes a simple software program so that users with computer access can easily calculate strength of relationships between MSD risk and the various hazard scores, in order to identify those hazards having the greatest effect on MSD risk for workers in that particular job. This information is then used in participative processes to identify job-specific causes and possible solutions, and to prioritise risk control interventions.

### More general implications for workplace management

In many OHS jurisdictions the workplace owner or most senior manager has ultimate responsibility for eliminating or reducing OHS risks as far as is reasonably practicable. In medium and large workplaces, however, direct responsibility for MSD risk management is often delegated to technical or professional experts, leaving line managers with little direct involvement. This is problematic because the decisions and behaviours of supervisors and managers are the source of many hazards, particularly psychosocial ones, and technical experts typically lack the necessary authority to deal effectively with such issues. To achieve more effective MSD risk management, there appears to be a need to restructure some responsibilities so that OHS management responsibilities and procedures are more closely integrated with those of line managers.

Such changes also have implications for the professional competencies required of both line managers and OHS personnel. In fact the need for skill development related to ‘psychosocial risk’ was recognised by over 80 percent of senior managers and other respondents to a survey of EU stakeholders conducted as part of the PRIMA-EF project [72]. In light of the extent and high costs of MSDs, we suggest that the bodies responsible for maintenance of professional standards for OHS and general management professionals should treat this issue as urgent.

## Summary

Research evidence on the work-related causes of MSDs, which include both psychosocial and physical hazards, is not reflected in current workplace risk management practices and there are some major barriers to achieving more effective management. These barriers include the content of most regulatory and guidance documents targeting MSDs, which currently reflects the widespread but misguided belief that risk arises largely or entirely from physical hazard exposures, and the correspondingly narrow focus of MSD risk assessment tools on task-specific physical hazards. The conventional OHS risk management paradigm is also a barrier to change because it does not promote effective management of risks that have complex aetiologies entailing interactions between multiple, diverse hazards, such as is the case for MSDs.

It is argued that more effective workplace management of MSD risk requires a systems-based management framework and more holistic assessment and control procedures to address risk from all relevant hazards *together* rather than in isolation from each other. Accordingly, there is a need for risk management tools that address risk from psychosocial along with ‘manual handling’ hazards, in ways that support participation by workers themselves. The successful implementation of such changes will require closer integration of the roles of OHS personnel with those of line managers.

## Abbreviations

MSD: Musculoskeletal disorder
OHS: Occupational Health and Safety
DALYS: Disability adjusted life years
WHO: World Health Organisation
ILO: International Labour Organisation
PRIMA-EF: Psychosocial risk management – excellence framework
